# Arbuscular Mycorrhizal Fungal Community Composition in *Carludovica palmata, Costus scaber* and *Euterpe precatoria* from Weathered Oil Ponds in the Ecuadorian Amazon

**DOI:** 10.3389/fmicb.2017.02134

**Published:** 2017-11-07

**Authors:** Mónica Garcés-Ruiz, Carolina Senés-Guerrero, Stéphane Declerck, Sylvie Cranenbrouck

**Affiliations:** ^1^Laboratory of Mycology, Earth and Life Institute, Université catholique de Louvain, Louvain-la-Neuve, Belgium; ^2^Laboratorio de Micología, Facultad de Ciencias Exactas y Naturales, Pontificia Universidad Católica del Ecuador, Quito, Ecuador; ^3^Escuela de Ingeniería y Ciencias, Tecnologico de Monterrey, Monterrey, Mexico; ^4^Laboratory of Mycology, Mycothèque de l’Université catholique de Louvain (BCCM/MUCL^1^), Earth and Life Institute, Université catholique de Louvain, Louvain-la-Neuve, Belgium

**Keywords:** arbuscular mycorrhizal fungi, weathered crude oil pond, symbiosis, Amazon forest, Ecuador, community composition

## Abstract

Arbuscular mycorrhizal fungi (AMF) are ubiquitous to most natural and anthropized ecosystems, and are often found in polluted environments. However, their occurrence and community composition in highly weathered petroleum-polluted soils has been infrequently reported. In the present study, two ponds of weathered crude oil and their surrounding soil from the Charapa field in the Amazon region of Ecuador were selected and root colonization by AMF of their native plants investigated. The AMF community was further analyzed in three selected plant species (i.e., *Carludovica palmata, Costus scaber* and *Euterpe precatoria*) present in the two ponds and the surrounding soil. A fragment covering partial SSU, the whole ITS and partial LSU rDNA region was amplified (i.e., 1.5 kb), cloned and sequenced from the roots of each host species. AMF root colonization exceeded 56% in all plant species examined and no significant difference was observed between sites or plants. For AMF community analysis, a total of 138 AMF sequences were obtained and sorted into 32 OTUs based on clustering (threshold ≥97%) by OPTSIL. The found OTUs belonged to the genera *Rhizophagus* (22%), *Glomus* (31%), *Acaulospora* (25%) and *Archaeospora* (22%). *Glomus* and *Archaeospora* were always present regardless of the plant species or the site. *Acaulospora* was found in the three plant species and in the two ponds while *Rhizophagus* was revealed only in the surrounding soil in one plant species (*Euterpe precatoria*). Our study contributed to the molecular community composition of AMF and revealed an unexpected high presence of four AMF genera which have established a symbiosis with roots of native plants from the Amazon forest under high polluted soil conditions.

## Introduction

Ecuador is considered as a biodiversity hot spot, especially the Amazonian region which is emblematic for its rich and diverse flora and fauna. Despite of its ecological importance, the seventies marked the initiation of oil exploration and exploitation of the Amazonian forest. This led to a series of environmental disasters such as the contamination of rivers and groundwater, deforestation, precarious waste storage or weathered crude oil ponds (Armstrong and Vallejo, 1997).

Crude oil is a mixture of many compounds, such as alkanes, aromatics, asphaltenes and resins (Robertson et al., 2007). The alkanes and the polyaromatic hydrocarbons are the most easily biodegradable, by contrast to the asphaltenes and resins which are more resistant (Singh, 2006). The physicochemical composition affected by temperature, sun radiation, humidity and biological action left weathered crude oil accrued, eventually turning them in environmental liabilities (Singh, 2006).

The lack of environmental laws in Ecuador until 1990 (Armstrong and Vallejo, 1997) left around 2550 environmental liabilities caused by the oil industry (Ministerio del Ambiente de Ecuador [MAE], 2015). PetroAmazonas EP, an Ecuadorian public enterprise engaged in the exploration and extraction of oil reserves has concentrated its activities in production fields in the Amazon basin. Moreover, it is committed to the recovery of some environmental liabilities. Some of the weathered crude oil ponds are between 30 to 40 years old, despite of the intensive soil contamination, gradually these have been naturally recolonized by plants from the area.

The environmental consequences of weathered crude oil have mostly been measured in terms of flora and fauna, while microbial diversity has been most often ignored, even though microbes provide many ecological services.

Arbuscular mycorrhizal fungi (AMF) are soil inhabitants forming associations with the vast majority of plant species. They play key roles in soil processes (e.g., soil structure, biogeochemical cycles) (Rillig, 2004; Simard and Austin, 2010), help plants to acquire nutrients in exchange for carbohydrates (Abbott and Lumley, 2014) and protect them from biotic (Smith and Read, 2008) and abiotic (Plouznikoff et al., 2016) stresses. For instance, Cabello (1999) observed an increase in plant height and shoot biomass as well as P and Zn content in AMF-colonized *Medicago sativa* species grown in a hydrocarbon-polluted substrate as compared to non-colonized plants. Rajtor and Piotrowska-Seget (2016) argued that phytoremediation with AMF and/or hydrocarbon-degrading microorganisms are an effective strategy for dissipation of organic pollutants.

Arbuscular mycorrhizal fungi have been described in various biomes from temperate (Dodd, 2000) to tropical (Declerck et al., 1998; Homeier et al., 2013) and polar/boreal ecosystems (Öpik et al., 2013), under natural forests (Schüßler et al., 2015) as well as under crop cultivation systems (Senés-Guerrero et al., 2014; Buysens et al., 2016). A number of studies also reported their presence in environments polluted by heavy metals (Vallino et al., 2006; Liang et al., 2009; Gil-Cardeza et al., 2014; Wei et al., 2015; Ferrol et al., 2016) or petroleum hydrocarbons (Cabello, 1997; Hassan et al., 2014; de la Providencia et al., 2015). Although, their diversity in Ecuadorian petroleum hydrocarbons polluted soils is mostly unknown. Only one study (Villacrés et al., 2014) reported the presence of spores belonging to *Glomus* and *Acaulospora* in hydrocarbon-polluted soils from the Orellana province in the city La Joya de los Sachas (Amazon region). Species identification was done via morphological examination of spores which could highly change with age and due to the influence of different environmental stresses (Clapp et al., 1995; Bever et al., 1996; Declerck et al., 2000; Rillig et al., 2013).

Nowadays, molecular characterization is the current methodology that has reclassified a high number of AMF species (Souza, 2015). Krüger et al. (2012) developed a phylogenetic reference data for systematics and phylotaxonomy of AMF, that has been used as DNA barcode for AMF (Senés-Guerrero and Schüßler, 2016b; Wang et al., 2016). This reference dataset is an important tool to identify AMF from environmental samples. Interestingly, various surveys have been conducted on AMF diversity in industrial soils polluted by oil, using morpho-taxonomy (Cabello, 1997; Villacrés et al., 2014) and more importantly molecular tools (Hassan et al., 2014; de la Providencia et al., 2015; Iffis et al., 2016) thus increasing the accuracy of species identification. It is therefore interesting to study microbial communities associated with these plants and polluted sites, focusing in particular on AMF. Among these environmental liabilities is the Charapa field, in the province of Sucumbíos. The site is heavily contaminated with petroleum hydrocarbons. However, through the years many plants have recolonized these sites naturally.

The aim of this study was to analyze AMF root colonization in different plants species composing the ponds and surrounding soil and to determine the AMF community from three specific plants (*Carludovica palmata, Costus scaber* and *Euterpe precatoria*) present across the sites.

## Materials and Methods

### Sampling Location

The sampling site was located in the Amazonian region in the Province of Sucumbíos, Ecuador, in the environmental liability known as Charapa field at 309 masl. This area corresponds to a plant community known as Lowland Evergreen Forest (Palacios et al., 1999; Pérez, unpublished).

**FIGURE 1:**
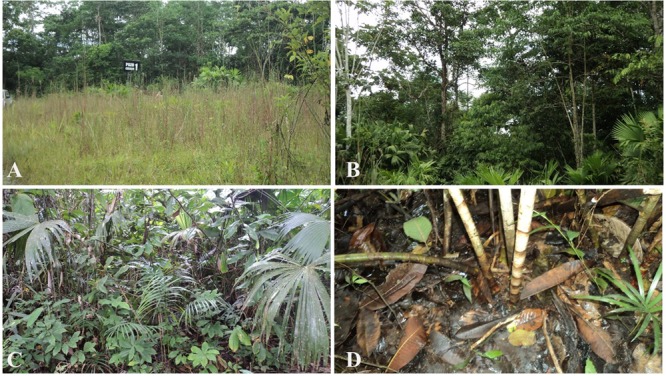
Charapa field. Vegetation present in the ponds and surrounding soil. **(A)** Herbaceous plants outside the ponds. **(B)** Trees present inside the ponds. **(C)** Herbaceous plants inside the ponds. **(D)** Contaminated soil where plants grow.

The site has a surface of 244 km^2^ and contains weathered crude oil polluted ponds. Two ponds were considered for this study. Pond 1 (76°48′57″ W, 00° 11′49″ S) has a surface of 330 m^2^ and Pond 2 (76°48′54″ W, 00°11′46″ S) a surface of 450 m^2^. The flora ecosystem was characterized by the botanist A. Pérez (unpublished). Both ponds are covered with a 10 cm layer of decomposed organic matter and are colonized by native plants from a secondary forest dominated by herbaceous plants and trees (**Figure 1**). In Pond 1, the predominant tree species are *Ficus insipida* (wild fig), *Ficus* cf. *americana* (West Indian laurel fig or Jamaican cherry fig), *Hieronyma alchorneoides* (*mascarey*) and *Croton lechleri* (dragon’s blood); while the more abundant herbaceous species are *Dimerocostus strobilaceus* (sour cane), *Carludovica palmata* (Panama hat plant –palm like), *Heliconia* cf. *chartacea* and several species of Araceae (i.e., *Euterpe precatoria* Mart. – palm like). In Pond 2, the predominant tree species are *Ficus insipida, Ficus* cf. *americana*, and several species of *Miconia*; while the dominant herbaceous species are *D. strobilaceus* (sour cane) and several species of *Costus* (sour cane – ginger like), *C. palmata, H.* cf. *chartacea* and several species of Marantaceae. In the surrounding soil the predominant tree species are *Ficus, Croton lechleri* and *Sapium glandulosum* while the dominant herbaceous species are *Costus scaber, Carludovica palmata, Heliconia* cf. *chartacea* several species of Araceae, besides pasture and crop species (i.e., cassava, banana cacao) were identified.

### Experimental Site

Herbaceous plants were randomly harvested from the two ponds and the surrounding soil in December 2012. Forty eight plants representing nine different species were sampled, (i.e., 21 in Ponds 1 and 2 and 6 in the surroundings) (**Table 1**) for root colonization evaluation. Due to the complexity of the sampling environment and the difficulties in the plant identification, three plant species (*Carludovica palmata, Costus scaber*, and *Euterpe precatoria*) found in the two ponds and surrounding soil, were further considered for AMF diversity assessment via molecular tools. For both analyses, the root samples were kept within the rhizosphere soil and dried at room temperature (28–30°C, standard temperature in the Amazon region) for 4 days and stored at 4°C. Before processing, roots were cleared from mineral and organic debris.

**Table 1:** Number of plant species sampled by site.

Plant species	Pond 1	Pond 2	Surrounding soil
*Monotagma* sp. 1	4	4	
*Polybotrya* sp. 2		1	1
*Geonoma* cf. *deversa* (Poit.) Kunth	1	2	
*Euterpe precatoria* Mart.	7	4	2
*Costus scaber* Ruiz & Pav.	2	1	1
*Costus pulverulentus* C. Presl	1	2	
*Costus lima* var. *scabremarginatus* Maas	2	3	1
*Carludovica palmata* Ruiz & Pav	2	3	1
*Polybotrya* sp. 1	2	1	

### AMF Root Colonization

Roots were cut into pieces of 2–3 cm and stained into acidic-blue ink as described by Walker (2005). The percentage of total colonization (%TC), arbuscular (%AC) and spores/vesicles (%VC) colonization were estimated under a dissecting microscope (Olympus BH2–RFCA, Japan) at 10x magnification according to McGonigle et al. (1990). An approximate of 100 intersections were observed per plant.

### DNA Extraction

Roots from *C. palmata, C. scaber* and *E. precatoria* were selected for AMF community composition. One plant per species was selected in each site and thus a total of 9 plants were analyzed. Between 55 and 100 mg of roots were grinded in liquid N_2_ with a mortar pestle to obtain a complete root disruption. The grounded material was transferred into the Lysing Matrix E tube from the FastDNA^®^ SPIN Kit for Soil (MP Biomedicals, United States) and DNA was extracted following the manufacturer’s protocol. DNA integrity was analyzed by electrophoresis using 5 μl of DNA stained with 100x GelRed^TM^ (Nucleic Acid Gel Stain, Biotium, Belgium). Samples were run at 100 V for 18 min in 0.5 x TAE buffer and stored at -20°C until further use.

### PCR, Cloning and Restriction Fragment Length Polymorphism (RFLP)

For AMF species delimitation, an extended DNA barcode region which comprises a part of the SSU rRNA gene, the complete ITS region (including the 5.8S rRNA gene) and approx. 800 bp of the LSU rRNA gene was amplified according to Krüger et al. (2009). The amplification requires a two-step PCR using AMF specific primers. For the first and nested PCR, the SSUmAf – LSUmAr and the SSUmCf – LSUmBr primer pairs were used, respectively. These primers have the widest taxon coverage when compared to other commonly used primers targeting a single nuclear rDNA marker (Kohout et al., 2014). Briefly, the reaction mix for both PCRs was done using the Phusion High Fidelity PCR Master Mix with HF Buffer (Thermo Fisher Scientific, Lithuania) with 0.5 μM as a final primer concentration for each primer (Sigma, Germany) and 0.2 μg mL^-1^ BSA (Albumin Bovine, AMRESCO, United States). Five μL of template DNA were used in 20 μL of final reaction. Thermal cycling was done in an Eppendorf Master-cycler Gradient (Eppendorf Nexus X2, Germany) with the following conditions for the first PCR: Five min. initial denaturation at 99°C; 40 cycles of 10 s. denaturation at 99°C, 30 s. annealing at 60°C and 1 min elongation at 72°C; and a 10 min. final elongation. In the nested PCR, 1 μL of the first PCR product was used as template in the same final reaction volume (20 μL), the thermal cycling conditions were the same as for the first PCR, except that the annealing temperature was 63°C and only 30 cycles were done. The amplification results in a single 1.5 kb fragment with species resolution power (Stockinger et al., 2010).

Cloning as well as RFLPs were as described by Krüger et al. (2009). In brief, after a nested PCR amplification, PCR products were loaded on 1% agarose gel to determine positive amplification (see above). The 1.5 kb fragments were cloned with the Zero Blunt TOPO PCR Cloning Kit (Invitrogen, United States) following the manufacturer’s protocol, with only one-third of the specified volume of all components used.

Forty eight clones from each cloning reaction were analyzed for correct length of plasmid inserts by colony-PCR using 1x GoTaq DNA Polymerase (Promega, United States) and M13F-M13R primers.

To detect variable sequences between the clones, RFLPs with two different enzymes were performed in 10 μL reaction volume, containing 5 μL colony-PCR product, with one of the restriction enzymes Hinf I (1 U), or RsaI (1 U) and the specific buffer (Roche, Germany).

Selected clones (e.g., 12–29 per cloning reaction) were purified using the Invisorb Spin Plasmid Mini Two kit (STRACTEC, Germany) according to the manufacturer’s protocol and sequenced using M13F-M13R primers at Macrogen Inc. (Korea).

### Sequence Assembly and Phylogenetic Analysis

Sequences were assembled and edited with SeqMan (Lasergene, Madison, WI, United States). The search for homologous sequences was conducted by blastn at the National Center for Biotechnology information (NCBI) website and Blast2GO^2^ (Conesa et al., 2005). Although a high-fidelity enzyme such as the Phusion DNA polymerase prevents chimera formation, sequences were checked manually to remove chimeric sequences. The AMF sequences were subsequently grouped into operational taxonomic units (OTUs) at a threshold of ≥97% sequence similarity, using the optimization of threshold-based linkage clustering runs from OPTSIL program (Goeker et al., 2009).

Nucleotide sequence alignments were performed with MAFFT version 7^3^ (Katoh and Standley, 2013), followed by manual adjustments using an AMF freely available reference alignment (Krüger et al., 2012)^4^ and environmental sequences from the NCBI platform at the Phylogenetic Data Editor (PhyDE)^5^.

A maximum-likelihood phylogenetic tree was assembled with 256 reference and environmental sequences and 32 single representative sequences from each OTU using RAxML-HPC2 (Stamatakis et al., 2008) on XSEDE ver. 8.2.9 on the CIPRES Science Gateway^6^ with 1000 bootstrap and the GTRGAMMA model (Krüger et al., 2012). Taxonomic annotations follow the classification of Schüßler and Walker (2010). All variant sequences obtained from the clone library were deposited at NCBI with accession numbers MF589988-MF590019.

### Diversity Analysis

Analysis of the community composition of AMF, in roots, was based on the number of clones (determined by analyzing the RFLP patterns) representing each AMF species (determined by phylogenetic analysis) present in the site and plant species. The Shannon diversity index (H′), was calculated using the vegan package (Oksanen et al., 2017) in R Development Core Team (2016). The Shannon diversity index (H′) was calculated using the diversity function by the formula H′ = -Σpi log(b) pi, where pi is the proportional abundance of AMF species or genera *i* and *b* is the base of the logarithm (Oksanen et al., 2017).

Principal component analysis (PCA) was used to analyze the similarity of the AMF community composition present in the plant species and the sites. Data were square root normalized and analyzed using the vegan package of R adjusting the total inertia to the number of variables.

### Statistical Analysis

Statistical analyses were performed using the IBM SPSS statistic 22 software. Data for AMF root colonization percentage were analyzed by one way ANOVA. Normal distribution was checked and non-normal data were normalized by arcsine transformation before analysis. One way ANOVA was used to determine significant difference between plant species and between sites AMF root colonization.

## Results

### AMF Root Colonization

**FIGURE 2:**
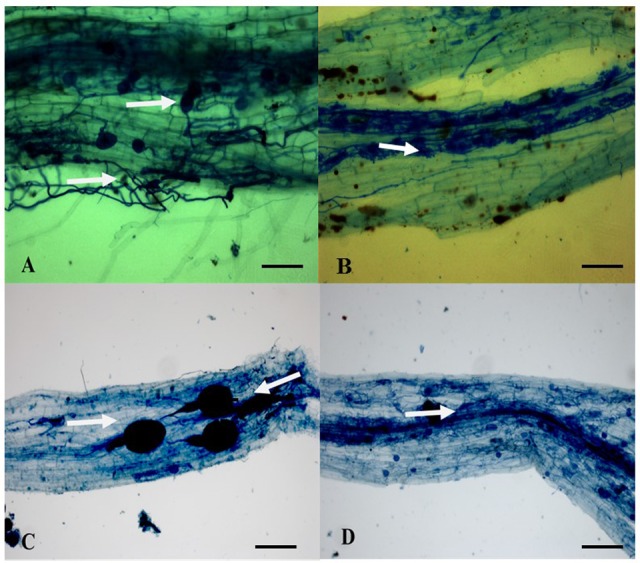
Ink staining of AMF root colonization (Walker, 2005). **(A)** Spores/vesicles (white arrow above), and hyphae (arrow below) in *Carludovica palmata*. **(B)** Arbuscules (white arrow) in *Costus scaber*. **(C)** Big spores/vesicles presenting a well -defined subtending hypha (white arrows) in *Polybotrya* sp. 2 **(D)** Hyphae (white arrow) in *Euterpe precatoria*. Scale bar **(A,B)** 300 μm, **(C,D)** 100 μm.

**FIGURE 3:**
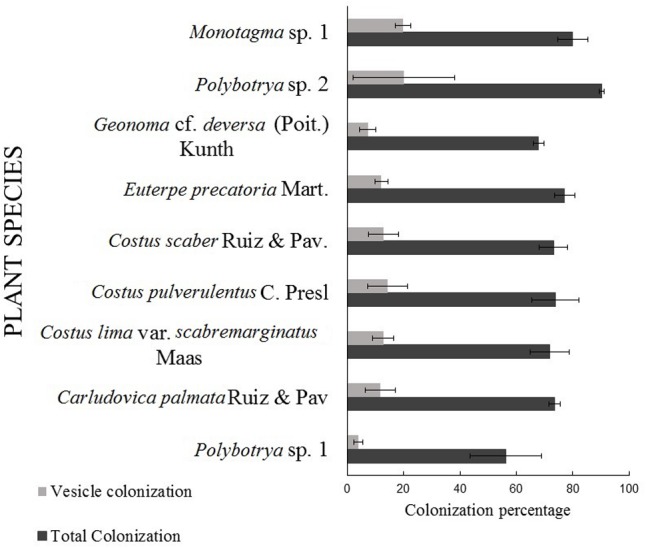
Arbuscular mycorrhizal fungi total and intraradical spores/vesicles colonization (McGonigle et al., 1990) of plant species present in Ponds 1, 2 and surrounding soil. Data represent mean values and standard errors (SE) of 8 (*Monotagma* sp. 1), 2 (*Polybotrya* sp. 2), 3 (*Geonoma* cf. *deversa*), 13 (*Euterpe precatoria.*), 4 (*Costus scaber*), 3 (*Costus pulverulentus*), 6 (*Costus lima*), 6 (*Carludovica palmata*), 2 (*Polybotrya* sp. 1) replicates. No significant differences were observed on %VC or %TC between plant species (*P* ≤ 0.05, Tukey HSD test).

Regardless of the origin of the sample, root colonization was observed on all nine plant species (Pond 1 and 2 and surrounding soil) (**Figure 2**). All the plants surveyed had a total colonization percentage (%TC) above 56% ± 12.7 and a total vesicle colonization percentage (%VC) between 4% ± 1.5 and 20% ± 18 (**Figure 3**). Arbuscules were observed only in six plants out of the 48 observed [i.e., 1 (*Costus pulverulentus*), 2 (*Costus scaber*), 1 (*Costus lima* var. *scabremarginatus*), 1 (*Carludovica palmata*) and 1 (*Monotagma* sp. 1)]. The %AC was between 1 and 12% (data not shown). Due to the presence of few representatives of some plant species and to preserve the plant diversity in the ponds, it was not always possible to sample the same plant species with the same number of replicate in the three sites (**Table 1**). However, no significant difference were observe in the %TC or %VC between plant species (*P* = 0.216 and *P* = 0.382, respectively) (**Figure 3**) and between sites (*P* = 0.495 and *P* = 0.284, respectively – data not shown).

### AMF Root Diversity

**FIGURE 4:**
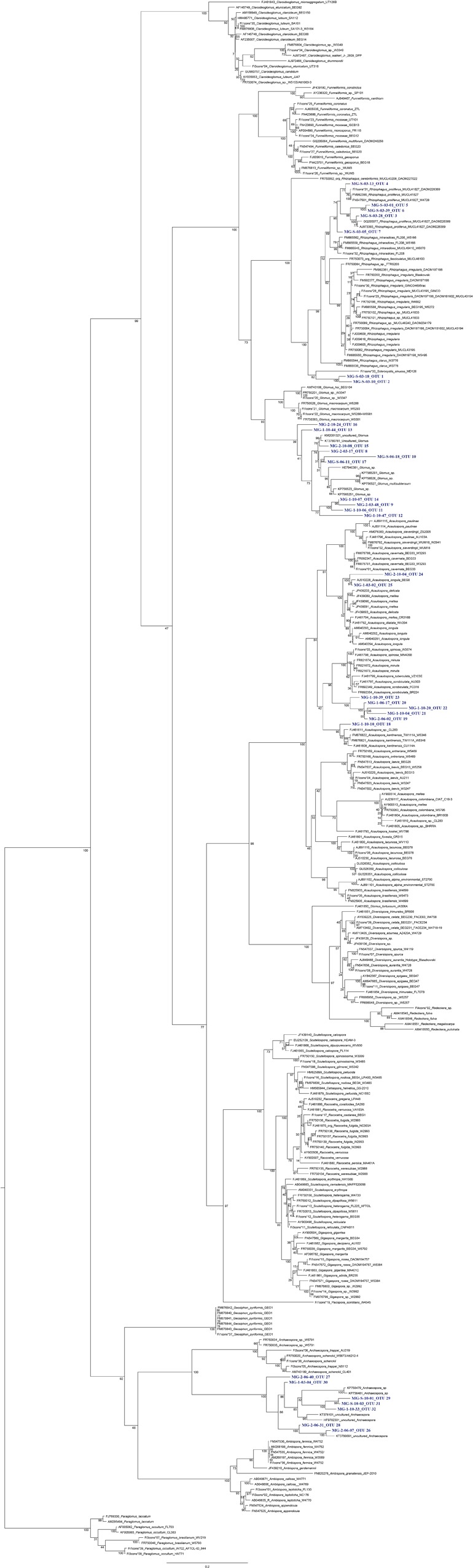
Maximum-likelihood phylogenetic tree based on the SSU-ITS-LSU rDNA region showing the distribution of the 19 OTUs obtained from the three sites (Pond 1, Pond 2 and surrounding soil) associated with the roots of 3 different plant species (*E. precatoria, C. scaber*, and *C. palmata*). Sequence data were analyzed in combination with the reference sequences from Krüger et al. (2012) and GenBank sequences. Bootstrap values (1000 replicates) are shown above the branches.

**FIGURE 5:**
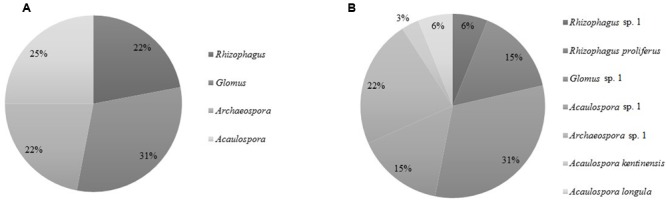
Occurrence of AMF Operational Taxonomic Units (OTUs) according to the **(A)** percentage of OTUs representing Glomeromycota genera, **(B)** percentage of OTUs representing Glomeromycota species independently of the site or plant species.

**FIGURE 6:**
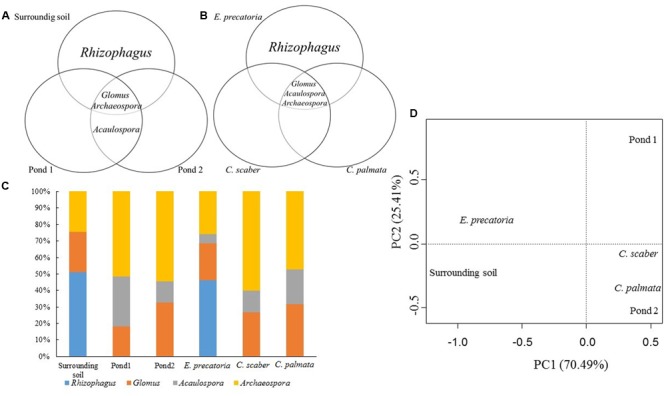
Distribution of the AMF genera according to the sites **(A)** and the three plant species **(B)**. Relative abundance distribution **(C)** and principal component analysis (PCA) of AMF genera **(D)**. Sites: oil ponds 1, 2 and surrounding soil. Plant species: *E. precatoria, C. scaber* and *C. palmata.*

The nested PCR amplified the SSU-ITS-LSU (1.5 kb) rDNA region from all selected root samples. The number of sequences amplified was 138. According to the clustering (threshold ≥97%) by OPTSIL the sequences were grouped in 32 OTUs (**Figure 4** and **Table 2**). Four AMF genera and seven species were detected in the roots of the three plant species examined by maximum-likelihood phylogeny. Based on homology in GenBank and the phylogenetic tree (**Figure 4**), the percentage of OTUs per AMF genus was 22% (7 OTUs) of *Rhizophagus*, 31% (10) of *Glomus*, 25% (8) of *Acaulospora* and 22% (7) of *Archaeospora* (**Figure 5A**). The genus *Acaulospora* was found in the three plant species in the two ponds but not in the surrounding soil. On the contrary, *Rhizophagus* was only observed in the surrounding soil specifically in *E. precatoria*. *Glomus* and *Archaeospora* were detected in all plant species in the three sites (**Figures 6A–C** and **Table 2**). According to AMF species, 5 OTUs (16%) belonged to *Rhizophagus proliferus* and *Acaulospora* sp. (minuta-scrobiculata-like), 2 (6%) belonged to *Rhizophagus sp.* and *Acaulospora longula*, 1 (3%) to *Acaulospora kentinensis*, 10 (31%) to *Glomus* sp., and 7 (22%) to *Archaeospora* sp. (**Figures 4, 5B**). The distribution was similar at the genus-level.

**Table 2:** Operational taxonomic unit (OTUs) identification according to the site and plant species.

Family	Genus	# OTU	OTU species ID	Site	Plant species
Glomeraceae	*Rhizophagus*	1	*Rhizophagus* sp. 1	Surrounding soil	*E. precatoria*
		2	*Rhizophagus* sp. 1	Surrounding soil	*E. precatoria*
		3	*R. proliferus*	Surrounding soil	*E. precatoria*
		4	*R. proliferus*	Surrounding soil	*E. precatoria*
		5	*R. proliferus*	Surrounding soil	*E. precatoria*
		6	*R. proliferus*	Surrounding soil	*E. precatoria*
		7	*R. proliferus*	Surrounding soil	*E. precatoria*
	*Glomus*	8	*Glomus* sp. 1	Pond 2	*E. precatoria*
		9	*Glomus* sp. 1	Pond 2	*E. precatoria*
		10	*Glomus* sp. 1	Surrounding soil	*C. scaber*
		11	*Glomus* sp. 1	Pond 1	*C. palmata*
		12	*Glomus* sp. 1	Pond 1	*C. palmata*
		13	*Glomus* sp. 1	Pond 1	*C. palmata*
		14	*Glomus* sp. 1	Pond 1	*C. palmata*
				Pond 2	*E. precatoria*
		15	*Glomus* sp. 1	Pond 2	*C. palmata*
		16	*Glomus* sp. 1	Pond 2	*C. palmata*
		17	*Glomus* sp. 1	Pond 2	*E. precatoria C. palmata*
				Surrounding soil	*C. scaber*
Acaulosporaceae	*Acaulospora*	18	*A. kentinensis*	Pond 1	*C. palmata*
		19	*Acaulospora* sp. 1	Pond 2	*C. scaber*
		20	*Acaulospora* sp. 1	Pon1, Pond 2	*C. scaber*
		21	*Acaulospora* sp. 1	Pond 1	*C. palmata*
		22	*Acaulospora* sp. 1	Pond 1	*C. palmata*
		23	*Acaulospora* sp. 1	Pond 1	*C. palmata*
		24	*A. longula*	Pond 2	*C. palmata*
		25	*A. longula*	Pond 1	*E. precaria, C. scaber*
				Pond 2	*C. palmata*
Archaeosporacea	*Archaeospora*	26	*Archaeospora* sp. 1	Pond 2	*C. scaber*
		27	*Archaeospora* sp. 1	Pond 2	*C. scaber*
		28	*Archaeospora* sp. 1	Pond 2	*C. scaber*
		29	*Archaeospora* sp. 1	Surrounding soil	*C. palmata*
		30	*Archaeospora* sp. 1	Pond 1 and 2	*E. precaria, C. scaber*
				Pond 1	*C. palmata*
		31	*Archaeospora* sp. 1	Surrounding soil, Pond 1	*C. palmata*
		32	*Archaeospora* sp. 1	Pond 1	*C. palmata*

**FIGURE 7:**
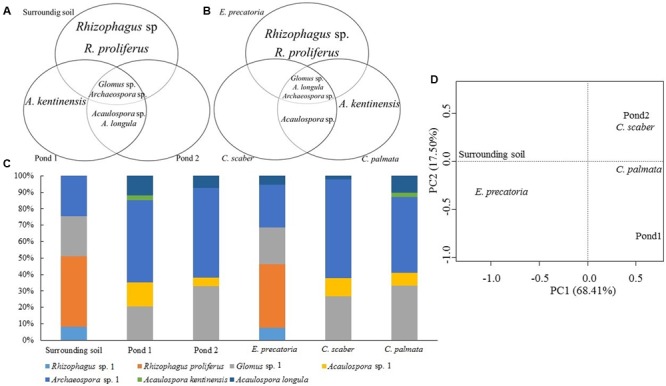
Distribution of the AMF species according to the sites **(A)** and the three plant species **(B)**. Relative abundance distribution **(C)** and principal component analysis (PCA) of AMF species **(D)**. Sites: oil ponds 1, 2 and surrounding soil. Plant species: *E. precatoria, C. scaber* and *C. palmata.*

Regardless of the site, the genera *Rhizophagus* sp. and *R. proliferus* were colonizing only *E. precatoria. A. kentinensis* was recorded only in *C. palmata*. *Acaulospora* sp. was shared between *C. palmata* and *C. scaber*. Finally *Glomus* sp., *A. longula* and *Archaeospora* sp. were revealed in the 3 plant species (**Figures 7A–C** and **Table 2**).

The PCA from the AMF community composition analyzed at the genus-level, demonstrated a similarity in the AMF community between *C. palmata, C. scaber* and Pond 2. Also similar are the communities present in *E. precatoria* and surrounding soil whereas Pond 1 was the most dissimilar (**Figure 6D**). A very similar pattern was observed when the AMF community composition was analyzed at the species-level (**Figure 7D**). Shannon diversity from AMF presented similar indices regardless of the site or plant species (**Table 3**).

**Table 3:** Values of AMF diversity (species and genera) by site and plant species according to Shannon’s index.

Plant species	# of plant samples	Pond 1		Pond 2		Surrounding soil		Shannon’s diversity index (plant species)	
		# of AMF species	# of AMF genera	# of AMF species	# of AMF genera	# of AMF species	# of AMF genera	AMF species	AMF genera
*E. precatoria*	3	2	2	2	2	2	1	1.40	1.20
*C. scaber*	3	3	2	2	2	1	1	0.99	0.93
*C. palmata*	3	4	3	2	2	1	1	1.25	1.05
*Shannon’s diversity index (sites)*		1.31	1.01	1.05	0.96	1.26	1.03		

## Discussion

Soils heavily contaminated by hydrocarbons are generally poor in plant and microbial diversity (Chibuike, 2013). Exploring AMF in hydrocarbon-polluted soils from natural environments (e.g., tropical rain forest) may provide unique information about the community composition and resilience of these root symbionts and their potentialities to protect plants from such stresses.

The presence of AMF in natural or polluted soils in the Amazonian basin of Ecuador has been poorly studied. The first report, to our knowledge, was made by Lunt and Hedger (1996) on the mycorrhizal colonization of trees in *Terra Firme* Cuyabeno, a wildlife reserve in the rainforest. They found that AMF colonization was the dominant mycorrhizal type but they did not study their diversity. Still today there are limited reports on AMF community composition in the Amazon region of Ecuador, contrary to the Amazon regions from Brazil, Colombia, Venezuela and Peru which have been much more explored (Janos and Sahley, 1995; Salamnaca and Silva, 1998; Peña-Venegas et al., 2006; Kalinhoff et al., 2009; Lopes Leal et al., 2009; Peña-Venegas, 2011; Stürmer and Siqueira, 2011).

Forty years ago, during the exploration of oil in the Charapa field, crude oil was spilled and accumulated in artificial ponds. Since then, a layer of organic matter consistently amassed allowing a variety of different trees and herbaceous plants to colonize and establish naturally. Total petroleum hydrocarbon (TPH) concentration inside the ponds and surrounding soil was high (i.e., above 5000 mg Kg^-1^ in Pond 1 and 2 and ∼1200 mg Kg^-1^ in the surrounding soil) (Personal communication by Centro de Servicios Ambientales y Químicos – PUCE). The presence of TPH in the surrounding soil suggested lixiviation from the ponds. Pollution was thus observed in the three sites, although four times higher in the two ponds as compared to the surrounding soil.

The thorough analysis of plant species in the ponds as well in the surrounding soil, demonstrated the systematic presence of AMF in roots with levels of colonization above 50% in each species analyzed. Root colonization was observed for the first time in *E. precatoria, C. scaber, C. palmata, Monotagma* sp.1, *Polybotrya* sp.2, *Geonoma* cf. *deversa, Costus pulverulentus, Costus lima*, and *Polybotrya* sp.1 isolated from oil polluted soils. Nonetheless AMF association in some of these genera has been already revealed, due to its presence in the rhizosphere on natural, agriculture or greenhouse conditions (Trufem, 1990; Santos et al., 2000; Wang and Qiu, 2006; Reyes-Jaramillo et al., 2008).

Numerous hyphae and vesicles were observed but curiously few or no arbuscules in agreement with the findings of Iffis et al. (2016). These authors recorded the presence of vesicles and hyphae but no arbuscules in the roots of three plants species (i.e., *Solidago canadensis, Populus balsamifera*, and *Lycopus europaeus*) growing in petroleum hydrocarbon wastes of three decantation basins from a former petrochemical plant, located on the south shore of the St-Lawrence River near Montreal (Canada). This very low or erratic occurrence of arbuscules could be attributed to several factors among which is the presence of pollutants as suggested by Cabello (1997). Interestingly, in soils contaminated by heavy metals, both Sonjak et al. (2009) and Maček et al. (2016) reported a low frequency or absence of arbuscules in the roots of plants naturally colonizing these soils.

An in-deep community analysis was conducted on *C. scaber, E. precatoria and C. palmata* because these plants are known to form associations with AMF (Janos, 1980; Santos et al., 2000; Wang and Qiu, 2006; Janos, 2007; Lopes Leal et al., 2009; Stürmer and Siqueira, 2011) and they were present in the two contiguous oil ponds and surrounding soil of the Charapa field study. To our knowledge, no information on AMF communities associated with these species exist. A number of AMF species belonging to *Acaulospora, Entrophospora* and *Glomus* were detected in an agroforestry system at the Upper Solimões River, at the heart of the Amazon region in Brazil, in which *E. precatoria* was present along with other plants known as regenerating species from secondary forest. Though no root colonization or AMF species diversity within roots was evaluated (Lopes Leal et al., 2009; Stürmer and Siqueira, 2011). Similarly, *C. palmata* (Janos, 1980, 2007) and *C. scaber* (Santos et al., 2000) are highly mycotrophic plants but the AMF communities in their roots remain undescribed.

Interestingly, from the DNA extraction of roots of the three plant species, four AMF genera (*Rhizophagus, Glomus, Acaulospora*, and *Archaeospora*) were detected. The taxonomic diversity at the genera level was low, although seven species within 138 AMF sequences were obtained for a total of 10, 8, 7 and 7 different OTUs within *Glomus, Acaulospora, Rhizophagus* and *Archaeospora*, respectively. Hassan et al. (2014) and de la Providencia et al. (2015) revealed a higher number of OTUs (27 and 21, respectively) in different hydrocarbon-polluted soils, although they analyzed a much larger number of sequences with a different molecular marker (69282 AMF 18S rDNA sequences from 454-pyrosequencing and 824 AMF 18S rDNA Sanger sequences).

The presence of *Archaeospora* was detected in 22% of the total number of OTUs. This family was present in the three sites and associated with all plant species analyzed. However, all the sequences were represented by unknown *Archaeospora* spp. (OTUs 24–32) in several well-resolved phylogenetic clades (**Figure 4**), suggesting the presence of new species. This genus was not reported when the 18S rDNA was sequenced in the studies of de la Providencia et al. (2015) and Iffis et al. (2016) on hydrocarbon-polluted soils. However Iffis et al. (2016) reported, in the same study, low abundance of unclassified Archaeosporaceae using the AMF ITS dataset. Similarly, Cabello (1997) and Villacrés et al. (2014) using classical spores taxonomy never described this genus in hydrocarbon-polluted soils. Hassan et al. (2014) and Iffis et al. (2014, 2016) recorded virtual taxa belonging to Archaeosporacea with a molecular approach in soils from a phytoremediation field trial at the site of a former petrochemical plant in Varennes and St-Lawrence River, Canada, respectively. Interestingly, Peña-Venegas (2011) have documented two species of *Archaeospora* (*Archaeospora leptoticha and Archaeospora trappei*) in the Amazonian region of Brazil and one in Colombia (*A. leptoticha*) demonstrating the presence of these genera in the Amazonian region. Further one phylotype belonged to this genus was recorded in roots of *Prunus persica* under two types of fertilization (inorganic, with or without manure) in a tropical agro-ecosystem of Venezuela (Alguacil Mdel et al., 2014).

The genus *Acaulospora* was found in 25% of the total number of OTUs. This genus was present in both ponds but not in the surrounding soil. Two OTUs belonged to *A. longula* (OTU 24 and 25) and 1 was related to *Acaulospora kentinensis* (OTU 18) and putatively unknown *Acaulospora* spp. (OTU 19–23), suggesting undescribed new species. The Acaulosporacea family has been defined as a stress-tolerant AMF due to its capacity to complete its life cycle with low biomass production that would thus reduce exposure to abiotic stress agents (Chagnon et al., 2013). This family was recorded in hydrocarbon-polluted soils with a predominance of 16% of the total number of species detected (Iffis et al., 2016). Villacrés et al. (2014) also noticed, by spores morpho-analysis, a predominance of *Acaulospora* as well as *Glomus* in hydrocarbon-polluted soils from the Orellana province in the Amazon region of Ecuador. Conversely, de la Providencia et al. (2015) obtained only a single OTU belonging to *Acaulospora*, associated with *Eleocharis obtusa* and *Panicum capillare*. Hassan et al. (2014) did not detect any *Acaulospora* spp. in Willow (*Salix* spp. L.) under hydrocarbon-polluted conditions. These suggest that the presence of *Acaulospora* spp. is not related to hydrocarbon contamination, but can be associated with the Ecuadorian Amazon region.

Finally, the predominance of *Glomus* (31%) with 10 different OTUs detected in the roots of the three plants species from the ponds and surrounding soil was in agreement with several studies that revealed the predominance of Glomeraceae in hydrocarbon-polluted soils (Cabello, 1997; Hassan et al., 2014; Villacrés et al., 2014; de la Providencia et al., 2015). The *Rhizophagus* genus was represented by 5 OTUs of *R. proliferus* (OTUs 3 to 7; synonymous of *Glomus proliferum*), 2 OTUs of *Rhizophagus* sp. closely related to *Sclerocystis sinuosa* (OTUs 1 and 2), revealing again the possibility of undescribed new species. Chagnon et al. (2013) determined that the Glomeraceae family has the capacity to colonize roots faster and that their extraradical hyphae abundance is higher than other AMF families (i.e., Acaulosporaceae, Gigasporaceae). This family is considered to have a ruderal life history, in agreement with its dominance in polluted environments. However, the OTUs belonging to the genus *Rhizophagus* were observed only in the surrounding soil in one plant species (*E. precatoria*). Conversely *Glomus* spp. were observed in all sites in all plant roots. Thus *E. precatoria* presented the most elevated percentage (58%) of OTUs compared to *C. scaber* (47%) and *C. palmata* (37%). *Rhizophagus* was dominant with 5 different OTUs according to the clustering (threshold ≥97%) by OPTSIL. Hassan et al. (2014) and de la Providencia et al. (2015) showed an elevated occurrence of *Rhizophagus* in hydrocarbon-polluted rhizosphere. Conversely de la Providencia et al. (2015) demonstrated that the AMF community structure in roots was totally different compared to the rhizosphere in the sediments associated with *E. obtusa* and *P. capillare*, where just one OTU belonging to the *Rhizophagus* genus was recorded.

These three AMF families (i.e., Glomeraceae, Acaulosporaceae, and Archaeosporaceae) have also been recorded associated with plants in the tropical mountain rain forest in the San Francisco River, Cordillera Real, in the south of Ecuador after reforestation experiments with local trees species (*Cedrela montana* Moritz ex Turcz., *Heliocarpus americanus* L., *Juglans neotropica* Diels and *Tabebuia chrysantha* (Jacq.) G. Nicholson) where 40 year old abandoned pastures were compared with old trees from the nearest pristine forest (Haug et al., 2010). Moreover, in potato crops in the Andean region of Ecuador, the same genera as the ones found in our study were detected and several *Acaulospora* spp. were recorded as the main root colonizers (Senés-Guerrero and Schüßler, 2016a). These results suggest a prevalence of these families in different environmental conditions in Ecuador. Thus, these AMF families could be considered as widely distributed and adapted to an extensive range of host plants and soil conditions in Ecuador. Indeed, a global assessment of AMF diversity revealed a high number of AMF taxa shared between continents (Davison et al., 2015), suggesting that dispersal mechanism such as wind, animals and human activities are responsible of their distribution in all kind of vegetative communities.

Additionally, results from de la Providencia et al. (2015) and our results showed the presence of almost all families of Glomeromycota with exception of Ambisporaceae, Geosiphonaceae, and Pacisporaceae in oil-polluted soils with abundance varying depending on environmental conditions (rainforest) as well as on the levels of pollution and native flora. Suggesting, one again, the world-wide distribution of these three families.

For the first time, AMF community composition was studied in crude oil ponds in a natural environment from the Amazon basin of Ecuador via molecular tools. Roots from several native plants species were highly colonized and diverse communities of AMF belonging to *Glomus, Rhizophagus, Archaeospora* and *Acaulospora* were associated with *C. scaber, E. precaria* and *C. palmata.* Seventy four percent of OTUs could not be ascribed to an existing AMF species suggesting the presence of a high number of potential new species. This is in agreement with global AMF molecular surveys where many unknown species are living in unstudied areas (Kivlin et al., 2011; Öpik et al., 2013) such as the Tibetan Plateau (Li et al., 2015) and the Andean region (Senés-Guerrero et al., 2014), suggesting that the diversity within this group of fungi is still underestimated. The unexpectedly high percentage of AMF colonization of the roots from plants growing in the hydrocarbon-polluted ponds of the Charapa field, further suggested that the AMF taxa found were able to adapt to these harsh conditions, representing potentially interesting strains for plant establishment and remediation of polluted soils.

## Ethics statement

Permits were given by the public enterprise PetroAmazonas EP for sampling and field study. The field study did not involve endangered or protected species.

## Author Contributions

MG-R: sampling, development of samples analysis, data collection, data analysis, interpretation of data. Drafting the work, commentaries corrections, final approval and agreement with all aspects of the work. CS-G: contribution to the analysis and interpretation of the data, draft correction and final approval and agreement with all aspects of the work. SD: contributions to analysis of the results, draft corrections final approval and agreement with all aspects of the work. SC: substantial contributions from conception to data analysis, draft correction and final approval and agreement with all aspects of the work.

## Conflict of Interest Statement

The authors declare that the research was conducted in the absence of any commercial or financial relationships that could be construed as a potential conflict of interest.

## Footnotes

2 https://www.blast2go.com/b2ghome

3 http://mafft.cbrc.jp/alignment/software

4 http://www.amf-phylogeny.com

5 http://www.phyde.de/

6 http://www.phylo.org
